# TCRcost: a deep learning model utilizing TCR 3D structure for enhanced of TCR–peptide binding 

**DOI:** 10.3389/fgene.2024.1346784

**Published:** 2024-10-02

**Authors:** Fan Li, Xinyang Qian, Xiaoyan Zhu, Xin Lai, Xuanping Zhang, Jiayin Wang

**Affiliations:** ^1^ School of Computer Science and Technology, Xi’an Jiaotong University, Xi’an, China; ^2^ Shaanxi Engineering Research Center of Medical and Health Big Data, Xi’an Jiaotong University, Xi’an, China

**Keywords:** systems immunology, T-cell receptor, peptide binding, prediction model, protein 3D structure, deep learning, 3D convolutional neural network

## Abstract

**Introduction:**

Predicting TCR–peptide binding is a complex and significant computational problem in systems immunology. During the past decade, a series of computational methods have been developed for better predicting TCR–peptide binding from amino acid sequences. However, the performance of sequence-based methods appears to have hit a bottleneck. Considering the 3D structures of TCR–peptide complexes, which provide much more information, could potentially lead to better prediction outcomes.

**Methods:**

In this study, we developed TCRcost, a deep learning method, to predict TCR–peptide binding by incorporating 3D structures. TCRcost overcomes two significant challenges: acquiring a sufficient number of high-quality TCR–peptide structures and effectively extracting information from these structures for binding prediction. TCRcost corrects TCR 3D structures generated by protein structure tools, significantly extending the available datasets. The main and side chains of a TCR structure are separately corrected using a long short-term memory (LSTM) model. This approach prevents interference between the chains and accurately extracts interactions among both adjacent and global atoms. A 3D convolutional neural network (CNN) is designed to extract the atomic features relevant to TCR–peptide binding. The spatial features extracted by the 3DCNN are then processed through a fully connected layer to estimate the probability of TCR–peptide binding.

**Results:**

Test results demonstrated that predicting TCR–peptide binding from 3D TCR structures is both efficient and highly accurate with an average accuracy of 0.974 on precise structures. Furthermore, the average accuracy on corrected structures was 0.762, significantly higher than the average accuracy of 0.375 on uncorrected original structures. Additionally, the average root mean square distance (RMSD) to precise structures was significantly reduced from 12.753 Å for predicted structures to 8.785 Å for corrected structures.

**Discussion:**

Thus, utilizing structural information of TCR–peptide complexes is a promising approach to improve the accuracy of binding predictions.

## 1 Introduction

T lymphocytes (T cells) play a critical role in the adaptive immune system (Marshall et al., 2018; Weber et al., 2021). T-cell receptors (TCRs) specifically identify antigenic peptides presented by major histocompatibility complexes (MHCs), initiating an immune response (La Gruta et al., 2018; Rudolph et al., 2006; Davis and Bjorkman, 1988). Predicting TCR–peptide binding is a fundamental computational challenge in systems immunology, which is crucial for drug development and immunotherapy design (Greiff et al., 2020; Winge-Main et al., 2020; Grazioli et al., 2023).

High-throughput immune repertoire sequencing has led to several publicly available, immune-related TCR sequence databases, such as VDJdb (Shugay et al., 2018), McPAS-TCR (Tickotsky et al., 2017), and IEDB (Mahajan et al., 2018), each containing hundreds to thousands of TCR–peptide amino acid sequence pairs. Using data from these databases, various computational models for TCR–peptide binding prediction have been developed, such as NetTCR-2.0, AttnTAP, TITAN, AVIB, and TCRPrediction, most of which extract specific features from the sequence pairs (Montemurro et al., 2021; Xu et al., 2022a; Weber et al., 2021; Grazioli et al., 2023; Koyama et al., 2023). The main differences between these models lie in how they encode the sequences and the types of deep learning models employed (Henikoff and Henikoff, 1992; Zhang et al., 2023; Pham et al., 2023). However, most methods predicted binding based on sequence information and did not implement the spatial information (structure of TCR–peptide binding). Structural data often provide critical insights that sequence data alone cannot, such as the spatial arrangement of atoms and interactions (Ovchinnikov and Huang, 2021). For instance, structural data can offer a more accurate description of atom pairs that are spatially close but distant in the linear sequence. Structural information has been shown to improve the performance in other areas of protein research, such as in predicting protein–protein interactions, with methods like SGPPI (Huang et al., 2023), PCA-Pred (Siva Shanmugam et al., 2021), and PSG-BAR (Pandey et al., 2022) demonstrating superior results. Therefore, we aim to incorporate TCR–peptide structures into our models to improve the accuracy of binding prediction.

Obtaining a large amount of TCR–peptide structures remains a great challenge. The number of available TCR–peptide structures is significantly lower than the number of sequences. Only a few hundred validated TCR–peptide structures are currently available (Zvyagin et al., 2020; Jisna and Jayaraj, 2021). These structures can be accurately determined using experimental techniques, such as X-ray crystallography, nuclear magnetic resonance spectroscopy, and electron microscopy. However, these methods are labor-intensive and time-consuming (Jisna and Jayaraj, 2021).

The rapid development of AI-based protein structure prediction methods makes it possible to determine 3D protein structures from amino acid sequences (Zhang et al., 2021; Shokrani et al., 2023). These approaches have demonstrated high accuracy and reliability in predicting protein structures (Eswar et al., 2006; Song et al., 2013; Roy et al., 2010; Das and Baker, 2008). For example, AlphaFold2, an advanced end-to-end algorithm based on deep learning, can predict structures with atomic-level accuracy (Jumper et al., 2021). A recent advancement in protein structure prediction, using AlphaFold Multimer, has been released to address the prediction of multimeric protein complexes (Evans et al., 2021). These methods comprehensively extract information from amino acid sequences, multiple sequence alignments, homologous structures, co-evolution signals, and other relevant data sources (Jones and Thornton, 2022). Recent research studies, including OmegaFold (Wu et al., 2022) and trRosettaX-Single (Wang et al., 2022), suggest leveraging large-scale natural language processing models to extract additional information relevant to protein structure prediction.

In this study, we focused on the CDR3A, CDR3B, and peptide regions because CDR3 loops are primarily responsible for interacting with peptides (Joglekar and Li, 2021; Chiffelle et al., 2020). We evaluated the performance of AlphaFold2 (Jumper et al., 2021) and OmegaFold (Wu et al., 2022) in predicting the structures of CDR3A, CDR3B, and peptide segments and found their performance to be comparable. For this study, we chose AlphaFold2 and used AlphaFold Multimer, which are specifically designed for predicting complex protein structures, to predict TCR–peptide interactions. We observed some inaccuracies in the predicted TCR–peptide structures, even though they adhered to established structural constraints. One possible reason is that the predictors often focus more on the main chains than on the side chains. These models primarily construct structures based on the main chains, with the side chains added later for fine-tuning (Jumper et al., 2021). However, binding structures involve interactions not only within the main and side chains but also between them (Chakrabarti and Pal, 2001). Therefore, to obtain high-quality structures for binding prediction, it is essential to apply a correction step to the predicted CDR3 structures.

Consequently, we developed TCRcost, a deep learning framework designed to predict TCR–peptide binding using corrected structures. We designed a correction model specifically to refine the accuracy of the predicted structures. This correction model takes into account both the main and side chains, ensuring that their interactions are accurately represented. The model first corrects the main chain and the side chain independently and then integrates them into a single structure for final adjustments. TCRcost utilizes a 1D convolutional neural network (1DCNN) to capture relationships between neighboring atoms and a long short-term memory (LSTM) model to analyze global atomic interactions. TCRcost uses a 3D convolutional neural network (3DCNN) to extract information from the atomic characteristics and corrected 3D coordinates of each atom. A fully connected layer is then used to predict TCR–peptide binding. We evaluated the performance of TCRcost in terms of both the structure correction and binding prediction. TCRcost achieved high accuracy (0.974) for binding prediction. Corrected structures were more similar to precise structures, whose average root mean square distance (RMSD) to precise structures was much smaller (12.753 Å between predicted structures and precise structures and 8.785 Å between corrected structures and precise structures) (Kufareva and Abagyan, 2012). Additionally, the accuracy of binding prediction improved significantly, increasing from 0.375 to 0.762 when using corrected structures compared to the original predicted structures.

## 2 Methods

**FIGURE 1:**
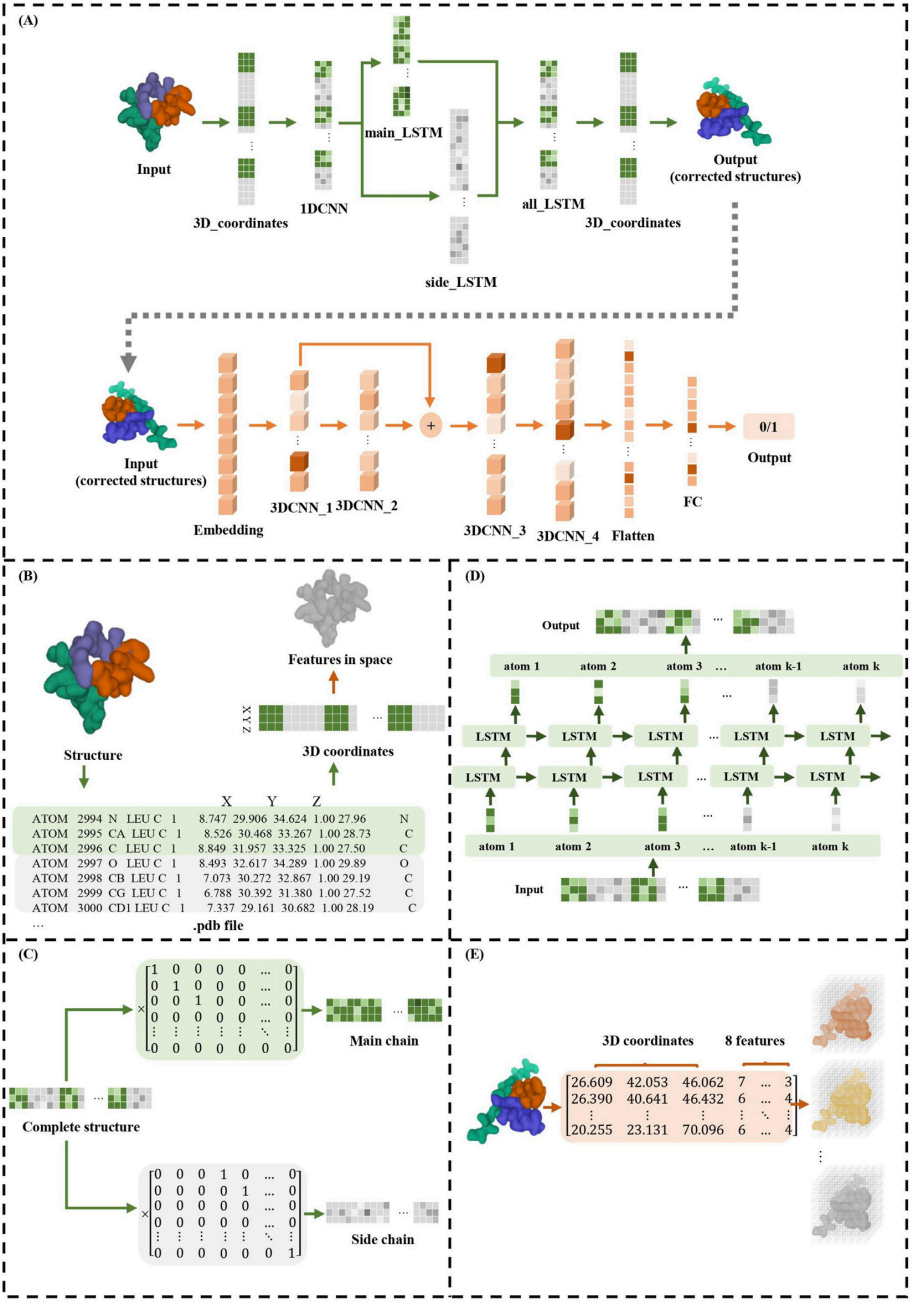
TCRcost corrects predicted structures and then predicts TCR–peptide binding. **(A)** The overall architecture of the TCRcost model. The 3D coordinates of the predicted structures with low quality were fed into the framework to accurately predict TCR–peptide binding. The correction module corrected the atoms of the main and side chains separately after capturing the relationships of adjacent atoms by 1DCNN, then combined them into entire structures, and processed them with LSTM (green area). The binding prediction module extracted features of the structures and predicted the likelihood of TCR–peptide binding by 3DCNN and MLP (orange area). **(B)** The transformations of different representations of TCR–peptide structures. **(C)** The separation of atoms into main chains and side chains. **(D)** The two-layer LSTM model of all_LSTM, which was the same as side_LSTM and main_LSTM. **(E)** The structures represented by 3D coordinates and eight atomic features were embedded into eight channels and fed into the 3DCNN model.

TCRcost consists of two modules: the structure correction module and the binding prediction module (Figure 1). In the correction module, it adjusts the spatial location for every atom. First, for each atom, the 1DCNN extracts the relationships between the neighboring atoms. Then, the atoms in the main and side chains are corrected by an LSTM. Next, the two chains are combined into an entire structure, which is also corrected by an LSTM model. The corrected structures are used to predict TCR–peptide binding along with the atomic characteristics. The structures are processed by the 3DCNN and MLP models to predict TCR–peptide binding.

### 2.1 Structure correction module

The precise structures and predicted structures are aligned to keep the order of the atoms consistent, and then the 3D atomic coordinates are encoded into the tensors as the input. The maximum length of the input is set to 400, and the redundant parts are truncated. For the structures having fewer atoms, we completed them with 0 up to the maximum length. In addition, to acquire the atoms of the main and side chains, TCRcost defines the main matrix *Mm* and the side matrix *Ms*, whose dimensions are 400*150 and 400*400, respectively. For the main matrix *Mm*, if the *i*th atom in the input vector is the *j*th atom of the main chains, then *Mm*_*ij*_ is 1; otherwise, it is 0. A similar definition is applied to the side matrix *Ms*.

Since the neighboring atoms in the input tensors have significant influences on each other, TCRcost uses the 1DCNN model to learn the relationships between them first, with the output tensors sharing the same dimensions as the input tensors. The 3D coordinates of atoms are fed into 1DCNN as three channels.

Then, the tensors of the entire structures are multiplied by *Mm* and *Ms* to separate the atoms of the main chains and side chains, respectively (Figure 1C). Both the main-chain parts and the side-chain parts are processed using a two-layer LSTM model (Figure 1D) and produce the intermediate results *O*_*main*_ and *O*_*side*_, respectively. Based on the different effects of the main and side chains on entire structures and protein–protein interactions, loss functions *L*_*main*_ and *L*_*side*_ are designed for *O*_*main*_ and *O*_*side*_, respectively.

*L*_*main*_ is calculated as FAPE as proposed in AlphaFold2 (Jumper et al., 2021). Formula 1 shows the specific calculation for *L*_*main*_. For each alignment, it chooses the identical residue from precise structures and *O*_*main*_ and then uses its three atoms in the main chain (N, CA, and C) to generate a new coordinate system. The average distance between the corresponding atoms in *O*_*main*_ and the precise structures *R*_*main*_ is determined as the distance between the two structures in the new coordinate systems. The operations performed above are repeated for each residue. Therefore, the average distance between the two structures under various alignments can be calculated as the loss value.

$$L_{main}\left(O_{main},R_{main}\right)=\frac{1}{N^{2}}\sum_{i,j}\|T_{o,i}^{-1}O_{main,j}-T_{R,i}^{-1}R_{main,j}\|,$$ (1)

where *N* is the number of residues in the structure, $T_{o,i}^{-1}$ and $T_{R,i}^{-1}$ are transformation vectors to the new coordinate system, which are built by the *i*th residue’s atoms in *O*_*main*_ and *R*_*main*_, and $O_{main,j}$ and $R_{main,j}$ are the *j*th residues in *O*_*main*_ and *R*_*main*_, respectively.

*L*_*side*_ is dependent on the distance matrices of the atoms in the side chains and is calculated similarly to the local-distance difference test results (Mariani et al., 2013). The corresponding distance matrices of precise structures and *O*_*side*_ are named *Dr* and *Dc*, respectively. We set different distance error thresholds, e.g., 0.5, 1.0, 2.0, and 4.0 Å, and also set a 25% proportion for each of the four thresholds. The specific calculation is shown in Formula 2. In the matrix obtained by subtracting the matrix *Dr* from the matrix *Dc*, the more elements whose absolute values are less than the threshold, the lower the loss value, and the two structures present more similarities. The segment-wise calculation of the difference between the two structures is possible in this approach. Since atoms in the side chains can interact with those of non-adjacent residues, *L*_*side*_ is a suitable choice that can reveal the global interaction of atoms.

$$L_{side}\left(Dc,Dr\right)=\sum_{k=1}^{4}p_{k}\times \frac{\sum_{i<j}ReLu\left(\left|{Dr}_{ij}-{Dc}_{ij}\right|-t_{k}\right)}{\sum_{i<j}\left|{Dr}_{ij}-{Dc}_{ij}\right|},$$ (2)

where the distance thresholds *t*_*k*_ are set to 0.5, 1.0, 2.0, and 4.0 Å and *p*_*k*_ is 0.25.

Finally, in order to avoid the atoms of the main chain being too far away from the atoms of the side chain in a single residue, the 3D coordinates of entire structures are processed, which are composed of *O*_*main*_ and *O*_*side*_ by a two-layer LSTM, and the 3D coordinates of the entire structures *O*_*all*_ after correction are output. To measure the loss value of the final result, *L*_*all*_ is calculated using Formula 3. It consists of two parts: the first part is computed in the same way as *L*_*main*_ (Formula 1), while the second part uses the L1 norm to restrict the length and angle of the peptide bonds and to measure the rationality of structures (Schulz and Schirmer, 1979).

$$L_{all}=L_{main}\left(O_{all},R_{all}\right)+\sum_{i=1}^{N}\left|{\alpha c}_{i}-{\alpha r}_{i}\right|+\sum_{i=1}^{N}\left|{lc}_{i}-{lr}_{i}\right|,$$ (3)

where ${\alpha c}_{i}$ and ${\alpha r}_{i}$ are the bond angles of the *i*th residue in the corrected and precise structures and ${lc}_{i}$ and ${lr}_{i}$ are the bond lengths of the *i*th residue in the corrected and precise structures, respectively.

### 2.2 Binding prediction module

The eight characteristics of the atoms are extracted from the TCR–peptide structures using RDKit, including element type, charge information, whether the atom is an aromatic atom, whether the atom is on the ring, hybridization mode, explicit valence of the atom, implicit valence of the atom, and total valence of the atom. To generate the potential negative samples for binding prediction, we replaced the corresponding peptide in positive samples with other peptides. The TCR–peptide samples are stored in .hdf files. Each input sample comprises a list of atoms, which consists of their 3D coordinates and eight characteristics.

In this work, 3DCNN is used to process TCR–peptide structures since it can vividly describe the location and relationships among atoms. The TCR–peptide input structures are represented by the 3D coordinates and the eight atomic characteristics (Figure 1E). The dimensions of input tensors are 48*48*48*8, where the number of channels is 8, the voxel grid size in each axis is 48, and each voxel size is 1 Å.

The binding prediction module consists of four convolutional layers with one residual block (Figure 1). The residual block enables pass gradients to be passed to the next layers without nonlinear activation (He et al., 2016), and it can help model training. Batch normalization was used to normalize each feature output, and the ReLU activation was employed for nonlinearity.

### 2.3 Performance evaluation

The performance of the correction was evaluated by the RMSD over atoms between the precise TCR–peptide structures and the corrected structures (Kufareva and Abagyan, 2012). Formula 4 is the calculation of RMSD scores. A lower RMSD score indicates a greater similarity between the two structures.

$$RMSD=\sqrt{\frac{1}{N}\sum_{i=1}^{N}d_{i}^{2}},$$ (4)

where *N* is the number of atoms and *d*_*i*_ is the distance between two corresponding atoms in the precise and corrected structures.

We used accuracy (ACC), recall (REC), precision (PRE), F1 score (F1), and area under the receiver operating characteristic curve (AUC) as the criteria of performance evaluation on binding prediction. Formulas 5–8 are the calculation methods of ACC, REC, PRE and F1 scores.

$$ACC=\frac{TP+TN}{P+N}=\frac{TP+TN}{TP+FN+TN+FP},$$ (5)

$$REC=\frac{TP}{P}=\frac{TP}{TP+FN},$$ (6)

$$PRE=\frac{TP}{TP+FP},$$ (7)

$$F1=2\times \frac{PRE\times REC}{PRE+REC}.$$ (8)

## 3 Results

We examined the performance of TCRcost in terms of structure correction and binding prediction. We also illustrated the shortcomings of the existing protein structure predictor in predicting TCR–peptide structures.

### 3.1 Datasets

A total of 121 precise TCR–peptide structures in the Protein Data Bank (.pdb) format were downloaded from the ATLAS dataset (Rose et al., 2011; Borrman et al., 2017). Since the CDR3 region plays an important role in the recognition of antigens and CDR3 sequences are far more abundant than the entire TCR sequences, only CDR3A, CDR3B, and peptide segments were kept. When generating the predicted structures, the corresponding CDR3A: CDR3B: peptide sequences were given to AlphaFold2.

During the experiments, the training sets and test sets were randomly split, according to the ratio of 8:2, and the ratio of positive and negative samples of binding prediction was set to the ratio of 1:1. In addition, a five-fold cross-validation was conducted to assess the performance of TCRcost in the experiments.

### 3.2 Predicting structures by AlphaFold2

**FIGURE 2:**
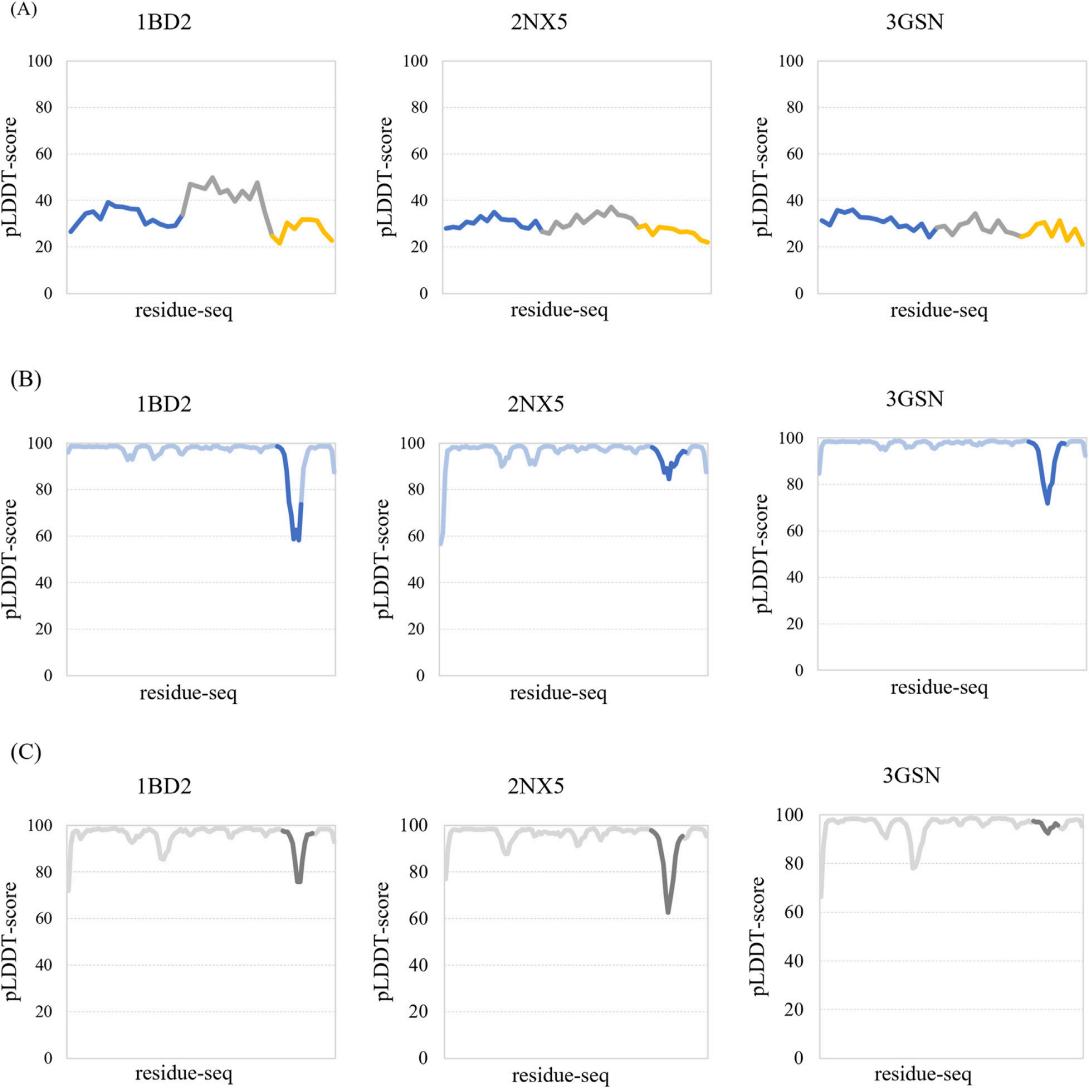
Performance of AlphaFold2 on TCR–peptide structure predictions. **(A)**The pLDDT score of the predicted structures of the complexes composed of CDR3B, CDR3A, and peptides, which are represented in blue, gray, and yellow, respectively. **(B)** The pLDDT score of the predicted structures of TCRB containing CDR3B. The dark blue areas correspond to the CDR3B regions. **(C)** The pLDDT scores of the predicted structures of TCRA containing CDR3A. The dark gray areas correspond to CDR3A regions.

We used AlphaFold2 to generate predicted TCR–peptide structures consisting of CDR3A: CDR3B: peptide segments. The quality of the predicted structure was assessed by using the predicted local-distance difference test (pLDDT) scores (Tunyasuvunakool et al., 2021). Since the pLDDT score of each residue was almost less than 50, the predicted TCR–peptide structures were more or less of low quality and low confidence (Figure 2A). Additionally, we predicted the entire TCRs, and AlphaFold2 showed lower pLDDT scores in CDR3 regions than in other adjacent regions (Figures 2B, C).

We also measured the degree of similarity between the predicted structures and the precise structures according to the RMSD scores. The average RMSD score was 12.753 Å, implying that some of them were significantly different. Compared to the performance on the main chains, AlphaFold2 performed worse on the side chains, as the average RMSD score was 11.379 Å for the main chains and the score was 13.411 Å for the side chains.

### 3.3 Corrected structures were more precise

#### 3.3.1 Ablation experiments

To test the potential advantages of processing the main chains and side chains independently, in terms of the accuracy of the correction model, we designed three models for comparison: LSTM_MAIN, LSTM_SIDE, and LSTM_ALL. LSTM_MAIN only processed the main chain atoms independently using *L*_*main*_ and *L*_*all*_ for training; LSTM_SIDE only processed the side-chain atoms independently using *L*_*side*_ and *L*_*all*_ for training; LSTM_ALL processed all atoms and only used *L*_*all*_ for training.

All models in the experiments were valid for correcting structures, and the RMSD scores decreased to varying degrees compared to the predicted structures (Table 1). The results revealed that LSTM_MAIN and LSTM_SIDE produced better intermediate results *O*_*main*_ and *O*_*side*_, respectively. TCRcost produced the best entire structures for the final results between them. It revealed that a preliminary correction was quite necessary for the main chains and side chains.

**TABLE 1:** Results of correction module ablation experiments measured by RMSD (Å).

	*O*_*main*_	*O*_*side*_	*O*_*all*_
AF_T	11.379	13.411	12.753
LSTM_ALL	—	—	9.183
LSTM_SIDE	—	11.002	8.945
LSTM_MAIN	9.296	—	8.957
TCRcost	9.925	11.049	8.785

AF_T represents the comparison between precise structures and predicted structures generated by AlphaFold2. The LSTM_ALL model processed all atoms together and only used *L*_*all*_ for training; the LSTM_SIDE model only processed the side-chain atoms independently using *L*_*side*_ and *L*_*all*_ for training; the LSTM_MAIN model only processed the main-chain atoms independently using *L*_*main*_ and *L*_*all*_ for training. *O*_*main*_ and *O*_*side*_ were the intermediate results comprising atoms in main chains and atoms in side chains, respectively, and *O*_*all*_ was the final result comprising all atoms in entire structures.

In the training process of the LSTM_ALL model, the unrelated atoms might interfere with each other, leading to a relatively low-quality correction. The LSTM_MAIN and LSTM_SIDE models could capture the precise prior knowledge of the main or side chains in the early stage. However, in the subsequent joint training process of all atoms, the other incorrect atoms that had not been initially corrected might affect the others, resulting in entire structures with relatively low quality. Getting more accurate distributions of the main and side-chain atoms was suggested to obtain better correction effects.

#### 3.3.2 Results of different models

We compared the performance of different kinds of models, including 1DCNN, 2D convolutional neural network (2DCNN), and LSTM. In the 1DCNN model, we set the 3D coordinates as three channels (features). We encoded the 3D coordinates of a TCR–peptide structure as a two-dimensional vector and then fed it into the 2DCNN model, which only contained one feature channel. TCRcost relied on the LSTM model.

The results showed that TCRcost outperformed the other two models (Table 2). For the intermediate results *O*_*main*_ and *O*_*side*_, the CNN sometimes performed slightly better than TCRcost. For the final results *O*_*all*_, LSTM was better than the others, as it clearly presented lower RMSD scores. In addition, the RMSD score of *O*_*all*_ was slightly higher than those of the *O*_*main*_ and *O*_*side*_ based on the CNN, but TCRcost showed the exact opposite results.

**TABLE 2:** Correction results of different models measured by RMSD (Å).

	*O*_*main*_	*O*_*side*_	*O*_*all*_
AF_T	11.379	13.411	12.753
1DCNN	9.384	10.441	10.462
2DCNN	9.304	10.586	10.593
TCRcost	9.925	11.049	8.785

AF_T represents the comparison between precise structures and predicted structures generated by AlphaFold2. 1DCNN , 2DCNN , and TCRcost models were constructed using 1DCNN, 2DCNN, and LSTM, respectively. *O*_*main*_ and *O*_*side*_ were the intermediate results comprising all main-chain atoms and all side-chain atoms, respectively, and *O*_*all*_ was the final result comprising all atoms in complete structures.

The CNN model was able to capture the relationships between adjacent atoms, which were greatly affected by the order of the input atoms. The LSTM model could capture the relationships between both the adjacent and non-adjacent atoms, and thus it achieved better correction effects. Since the majority of the relationships existed between the adjacent atoms, especially when the main chains and side chains were considered separately, the CNN and LSTM models presented similar performance. The positional relationships between all the atoms were difficult for the CNN models to learn, which lost the underlying global relationships, while the LSTM model could capture such relationships between pairs of atoms in the main chains and side chains, which occurred alternately throughout the entire structures. Thus, the LSTM model outperformed the CNN ones on the entire structure. Moreover, because of the folding and winding of the peptides in the TCR–peptide structure, there might be more cases where the atoms are spatially close but far apart in the sequence. The LSTM model was suggested as more suitable in this scenario.

#### 3.3.3 Results on different loss functions

In order to confirm the effectiveness of the designed loss functions, four experiments with different loss functions were carried out. Four different loss functions were set as follows: 1) MSE was used as the loss function that could measure the similarity between the corrected structure and the precise structure; 2) only *L*_*main*_ was used as the loss function; 3) only *L*_*side*_ was used as the loss function; 4) the loss functions were used in TCRcost.

For *O*_*all*_, the best correction was done using the TCRcost model, whereas the worst result was produced by using the MSE loss function (Table 3). L_main__model performed better on *O*_*main*_ and *O*_*side*_. However, due to the enormous computations of L_main_, the running times were much longer than those of the L_side_ and MSE runs. The TCRcost model ran faster than the L_main__model but obtained better corrections as well. By using L_main__model, L_side__model, and TCRcost, the RMSD scores of *O*_*all*_ were lower than those of *O*_*main*_ and *O*_*side*_. The RMSD score of *O*_*all*_ was higher than that of *O*_*main*_ by using the MSE_model.

**TABLE 3:** Correction results under different loss functions.

	*O*_*main*_ (Å)	*O*_*side*_ (Å)	*O*_*all*_ (Å)	Time (s)
AF_T	11.379	13.411	12.753	—
MSE_model	10.127	11.351	11.116	0.007
L_main__model	9.852	9.539	9.494	5.877
L_side__model	10.603	10.876	10.514	1.740
TCRcost	9.925	11.049	8.785	5.358

AF_T represents the comparison between precise structures and predicted structures generated by AlphaFold2. MSE_model, L_main__model, and L_side__model shared the same model architecture as TCRcost but had different loss functions during training, trained with the MSE loss function, the *L*_*main*_, and the *L*_*side*_, respectively. *O*_*main*_ and *O*_*side*_ were the intermediate results comprising all main-chain atoms and all side-chain atoms, respectively; *O*_*all*_ was the final result comprising all atoms in complete structures, and they were measured by RMSD (Å). In addition, “Time” was the time taken for one epoch during training, which was measured in seconds (s).

### 3.4 Corrected structures improved the accuracy of TCR–peptide binding prediction

First, we predicted TCR–peptide binding using the precise TCR–peptide structures. The ACC of the results was 0.947, which was higher than that of the methods based on the sequences only. The results demonstrated that TCR–peptide structures should contain more interaction-related information and could conduct better performances than the sequences in the binding prediction task.

**FIGURE 3:**
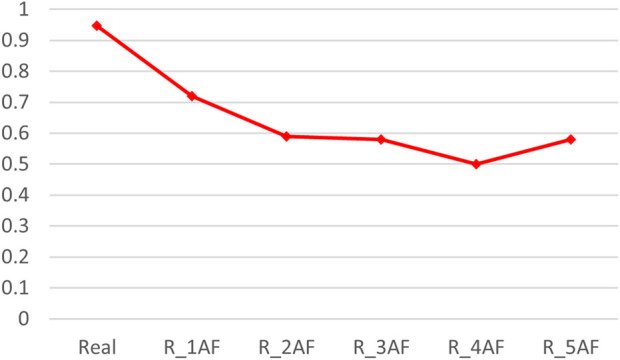
ACC scores of binding prediction using precise structures and predicted structures. “Real” denotes the dataset consisting of precise TCR–peptide structures, R_xAF is the dataset consisting of precise structures and predicted structures by AlphaFold2, and x indicates that the number of predicted structures is x times the number of accurate structures (x was 1, 2, 3, 4, and 5).

By adding varying amounts of the predicted structures (generated by AlphaFold2), the accuracy of the binding predictions decreased. The greater the number of predicted structures involved, the greater the decreases observed (Table 4). Comparing the accuracies between the models trained on the precise structures and the corresponding predicted structures, it was shown that the accuracy of binding predictions by the predicted structures was lower than that of the precise structures (Figure 3). These results suggest that, although significant improvements have been made in AI-based protein structure prediction, more work should be done in more nuanced areas.

**TABLE 4:** Binding prediction results of using the precise structures and predicted structures.

	Real	R_1AF	R_2AF	R_3AF	R_4AF	R_5AF
ACC	0.947	0.720	0.590	0.580	0.500	0.579
AUC	0.974	0.832	0.704	0.663	0.641	0.626
REC	1.000	0.707	0.525	0.642	0.650	0.661
PRE	0.905	0.725	0.604	0.571	0.512	0.567
F1	0.950	0.716	0.561	0.605	0.565	0.611

“Real” denotes the dataset consisting of precise TCR–peptide structures, R_xAF is the dataset consisting of precise structures and predicted structures by AlphaFold2, and x indicates that the number of predicted structures is x times the number of accurate structures (x was 1, 2, 3, 4, and 5). Abbreviations: ACC, accuracy; AUC, area under the receiver operating characteristic curve; REC, recall; PRE, precision; F1, F1 score.

By using the structure correction module, proposed in this work, it was possible to correct the predicted structures and produce corrected structures that were more similar to the precise structures. The performance of predicting the TCR–peptide binding using the corrected structures improved (Table 5). We randomly selected TCR–peptide sequences from the McPAS-TCR dataset, which were five times as many as the precise structures, and obtained corresponding predicted structures and corrected structures. In addition, we obtained the training and test sets from the corrected structures without the corresponding precise structures in the way described in Section 3.1. On the dataset of the corrected structures without the corresponding precise structures, the prediction accuracy was also improved and reached a level comparable to that of the dataset of the corrected structures with the precise structures.

**TABLE 5:** Binding prediction results using the predicted structures and corrected structures.

	R_AF	R_AF_COR	5AF	5AF_COR
ACC	0.375	0.762	0.475	0.760
AUC	0.379	0.762	0.445	0.760
REC	0.410	0.857	0.680	0.870
PRE	0.384	0.720	0.482	0.713
F1	0.396	0.783	0.564	0.784

R_AF and R_AF_COR are the datasets consisting of predicted structures and corrected structures, respectively, corresponding to precise TCR–peptide structures. 5AF and 5AF_COR were datasets consisting of predicted structures and corrected structures, respectively, which are without corresponding precise structures and were five times as many as precise structures. Abbreviations: ACC, accuracy; AUC, area under the receiver operating characteristic curve; REC, recall; PRE, precision; F1, F1 score.

The existing sequence-based methods, NetTCR-2.0 and TCRPrediction, which only predict the binding based on the sequences of the CDR3A, CDR3B, and peptide regions, were also involved in comparisons (Montemurro et al., 2021; Koyama et al., 2023). We compared TCRcost to NetTCR-2.0 and TCRPrediction on corrected structures and the corresponding residue sequences, respectively (Table 6). The TCRcost model was trained and tested on the dataset of the corrected structures without the corresponding precise structures. The models of NetTCR-2.0 and TCRPrediction were trained on the McPAS-TCR dataset and tested on the same dataset using the TCRcost model. TCRcost outperformed the other two sequence-based methods in TCR–peptide binding prediction. These results suggest that it is more accurate and feasible to use the structures, irrespective of how the existing models process the sequences.

**TABLE 6:** Binding prediction results of using TCR–peptide sequences and structures.

	ACC	AUC	REC	PRE	F1
NetTCR-2.0	0.667	0.640	0.792	0.641	0.708
TCRPrediction	0.701	0.786	0.850	0.661	0.744
TCRcost	0.760	0.760	0.870	0.713	0.784

Different TCR–peptide binding prediction methods were compared, among which NetTCR-2.0 and TCRPrediction are based on residue sequences, and TCRcost is based on corrected structures. Abbreviations: ACC, accuracy; AUC, area under the receiver operating characteristic curve; REC, recall; PRE, precision; F1, F1 score.

## 4 Discussion

Overall, TCRcost can predict TCR–peptide binding based on TCR–peptide structures and achieve better accuracy than predictions based on TCR–peptide sequences. Due to the scarcity of precise structures and the inaccuracy of the predicted structures by AlphaFold2, one contribution is to design the structure correction module. It obtains high-quality TCR–peptide structures from the original predicted structures. The loss function is designed based on the characteristics of the TCR–peptide structures, which guides the effective correction of the structures. With corrected structures, TCRcost accurately predicts binding by using the 3DCNN model. Here, we discuss the difficulties in obtaining TCR–peptide structures, the effectiveness of the loss functions, and the feasibility of using TCR–peptide structures for binding prediction.

Since the precise structures are limited, it is necessary to generate the structures using the AI-based protein structure predictors. We chose AlphaFold2 to predict TCR–peptide structures from TCR–peptide sequences. We only focused on CDR3A, CDR3B, and peptide segments. Due to the short length (11–18 residues) of the CDR3 region (Xu et al., 2022a), it is difficult for the deep learning model to obtain the information from adjacent residues. In addition, the CDR3 region has much higher diversity and variability, which further makes it harder to accurately predict the structure of the CDR3 region (Rudolph et al., 2006; Mora and Walczak, 2016). In the entire structure, the main chains and side chains can interfere with each other, and more operations are performed on the main chains when generating the predicted structures in AlphaFold2. These designs, we believe, should be the potential reasons leading to low quality on the entire predicted structures and differences in the quality between the main and side chains in predicted structures. Thus, we believe that the correction module, which corrects the main and side chains independently, is reasonable.

The loss functions in TCRcost are compared to several loss functions, including MSE, *L*_*main*_, and *L*_*side*_. MSE is a common loss function in machine learning. It presented the worst performance between the four loss functions. A possible reason is that MSE is too strict and inflexible, which greatly influences the rotation and translation of the structures. *L*_*main*_ should be a good measure of protein structure similarity without being affected by the protein rigidity transformation. However, the calculation of *L*_*main*_ is a difficult and costly task: it requires building a coordinate system centered on each residue in the protein and calculating the coordinates of each atom in that coordinate system. For a small number of atoms in main chains, *L*_*main*_ is an acceptable loss function. Instead of focusing on specific position coordinates, *L*_*side*_ is more concerned with the relative distance between atoms. The number of atoms in side chains is quite large, and their distances from each other are greatly influenced by protein interactions. During the training process, *L*_*side*_ globally captures the features of side chains and is independent of the rigid transformation of structures. *L*_*all*_ integrates *L*_*main*_ and adds restrictions on structures to ensure rationality, which works for the entire structure.

The prediction performance has demonstrated the advantages of using structure information. The 3DCNN model effectively describes the structures and is able to extract useful information. However, due to the limited confidence in the predicted structures, the binding prediction model should be designed to be robust to the quality of the TCR–peptide structures. The structure correction module has the potential to handle the various qualities of structures and obtain stable performance on the predictions. A possible idea is to train the model to learn the commonality in TCR–peptide structures. Further work should be considered and improve the deep learning models by integrating a noise-tolerant learning framework.

## 5 Conclusion

TCR–peptide binding prediction is an important computational problem to solve, but it is still extremely challenging because of the diversity of TCRs, the highly cross-reactive TCRs, and peptides. Many deep learning methods extracted information from sequences (Grazioli et al., 2023; Weber et al., 2021; Xu et al., 2022a; Xu et al., 2022b), but these pieces of information were limited. Since the application of protein structures enhanced the prediction of protein–protein interaction, we suggest that predicting TCR–peptide binding based on structures would improve the accuracy even more. Some existing issues, including the lack of TCR–peptide structure data, the imprecision of protein structure predicting tools, and the inapplicability of existing binding prediction models to structure data, led us to develop this framework TCRcost. To obtain enough structures with high quality, we proposed a structure correction module, which is able to correct the structures generated by AI-based structure predictors. We designed a binding prediction module to extract information from the corrected structures and predict TCR–peptide binding accurately. To the best of our knowledge, TCRcost is one of the first methods to accurately predict TCR–peptide binding from protein structures. We also propose to further integrate the information from both TCR–peptide sequences and their structures. A multi-modal model may be suitable for solving this scenario. In addition, the AI-based protein structure predictor should further consider the specific scenario, such as CDR3 loops, to better meet the clinical needs.

## Data Availability

The original contributions presented in the study are included in the article/Supplementary Material, further inquiries can be directed to the corresponding authors.

## References

[B1] Borrman T. Cimons J. Cosiano M. Purcaro M. Pierce B. G. Baker B. M. (2017). ATLAS: a database linking binding affinities with structures for wild-type and mutant TCR-pMHC complexes. Proteins 85, 908–916. 10.1002/prot.25260 28160322 PMC5860664

[B2] Chakrabarti P. Pal D. (2001). The interrelationships of side-chain and main-chain conformations in proteins. Prog. biophysics Mol. Biol. 76 (1–2), 1–102. 10.1016/s0079-6107(01)00005-0 11389934

[B3] Chiffelle J. Genolet R. Perez M. A. Coukos G. Zoete V. Harari A. (2020). T-cell repertoire analysis and metrics of diversity and clonality. Curr. Opin. Biotechnol. 65, 284–295. 10.1016/j.copbio.2020.07.010 32889231

[B4] Das R. Baker D. (2008). Macromolecular modeling with rosetta. Annu. Rev. Biochem. 77, 363–382. 10.1146/annurev.biochem.77.062906.171838 18410248

[B5] Davis M. M. Bjorkman P. J. (1988). T-cell antigen receptor genes and T-cell recognition. Nature 334 (6181), 395–402. 10.1038/334395a0 3043226

[B6] Eswar N. Webb B. Marti-Renom M. A. Madhusudhan M. S. Eramian D. Shen M. Y. (2006). Comparative protein structure modeling using Modeller. Curr. Protoc. Bioinforma. Chapter 5, 5–6. 10.1002/0471250953.bi0506s15 PMC4186674 18428767

[B7] Evans R. O’Neill M. Pritzel A. Antropova N. Senior A. Green T. (2021). Protein complex prediction with AlphaFold multimer. bioRxiv. 10.1101/2021.10.04.463034

[B8] Grazioli F. Machart P. Mösch A. Li K. Castorina L. V. Pfeifer N. (2023). Attentive variational information bottleneck for TCR-peptide interaction prediction. Bioinforma. Oxf. Engl. 39 (1), btac820. 10.1093/bioinformatics/btac820 PMC9825246 36571499

[B9] Greiff V. Yaari G. Cowell L. (2020). Mining adaptive immune receptor repertoires for biological and clinical information using machine learning. Curr. Opin. Syst. Biol. 24, 109–119. 10.1016/j.coisb.2020.10.010

[B10] He K. Zhang X. Ren S. Sun J. (2016). “Deep residual learning for image recognition,” in 2016 IEEE Conference on Computer Vision and Pattern Recognition (CVPR), Las Vegas, NV, June 27–30, 2016 (IEEE), 770–778.

[B11] Henikoff S. Henikoff J. G. (1992). Amino acid substitution matrices from protein blocks. Proc. Natl. Acad. Sci. U. S. A. 89 (22), 10915–10919. 10.1073/pnas.89.22.10915 1438297 PMC50453

[B12] Huang Y. Wuchty S. Zhou Y. Zhang Z. (2023). SGPPI: structure-aware prediction of protein-protein interactions in rigorous conditions with graph convolutional network. Briefings Bioinforma. 24 (2), bbad020. 10.1093/bib/bbad020 36682013

[B13] Jisna V. A. Jayaraj P. B. (2021). Protein structure prediction: conventional and deep learning perspectives. protein J. 40 (4), 522–544. 10.1007/s10930-021-10003-y 34050498

[B14] Joglekar A. V. Li G. (2021). T cell antigen discovery. Nat. methods 18 (8), 873–880. 10.1038/s41592-020-0867-z 32632239

[B15] Jones D. T. Thornton J. M. (2022). The impact of AlphaFold2 one year on. Nat. Methods 19, 15–20. 10.1038/s41592-021-01365-3 35017725

[B16] Jumper J. Evans R. Pritzel A. Green T. Figurnov M. Ronneberger O. (2021). Highly accurate protein structure prediction with AlphaFold. Nature 596, 583–589. 10.1038/s41586-021-03819-2 34265844 PMC8371605

[B17] Koyama K. Hashimoto K. Nagao C. Mizuguchi K. (2023). Attention network for predicting T-cell receptor-peptide binding can associate attention with interpretable protein structural properties. Front. Bioinform 3, 1274599. 10.3389/fbinf.2023.1274599 38170146 PMC10759225

[B18] Kufareva I. Abagyan R. (2012). Methods of protein structure comparison. Clifton, N.J. 857, 231–257. 10.1007/978-1-61779-588-6_10 PMC4321859 22323224

[B19] La Gruta N. L. Gras S. Daley S. R. Thomas P. G. Rossjohn J. (2018). Understanding the drivers of MHC restriction of T cell receptors. Nat. Rev. Immunol. 18 (7), 467–478. 10.1038/s41577-018-0007-5 29636542

[B20] Mahajan S. Vita R. Shackelford D. Lane J. Schulten V. Zarebski L. (2018). Epitope specific antibodies and T cell receptors in the immune epitope database. Front. Immunol. 9, 2688. 10.3389/fimmu.2018.02688 30515166 PMC6255941

[B21] Mariani V. Biasini M. Barbato A. Schwede T. (2013). lDDT: a local superposition-free score for comparing protein structures and models using distance difference tests. Bioinformatics 29 (21), 2722–2728. 10.1093/bioinformatics/btt473 23986568 PMC3799472

[B22] Marshall J. S. Warrington R. Watson W. Kim H. L. (2018). An introduction to immunology and immunopathology. Allergy, asthma, Clin. Immunol. official J. Can. Soc. Allergy Clin. Immunol. 14 (Suppl. 2), 49. 10.1186/s13223-018-0278-1 PMC6156898 30263032

[B23] Montemurro A. Schuster V. Povlsen H. R. Bentzen A. K. Jurtz V. Chronister W. D. (2021). NetTCR-2.0 enables accurate prediction of TCR-peptide binding by using paired TCRα and β sequence data. Commun. Biol. 4 (1), 1060. 10.1038/s42003-021-02610-3 34508155 PMC8433451

[B24] Mora T. Walczak A. M. (2016). Quantifying lymphocyte receptor diversity. bioRxiv [Preprint]. 10.48550/arXiv.1604.00487

[B25] Ovchinnikov S. Huang P. S. (2021). Structure-based protein design with deep learning. Curr. Opin. Chem. Biol. 65, 136–144. 10.1016/j.cbpa.2021.08.004 34547592 PMC8671290

[B26] Pandey M. Radaeva M. Mslati H. Garland O. Fernandez M. Ester M. (2022). Ligand binding prediction using protein structure graphs and residual graph attention networks. Molecules 27, 5114. 10.3390/molecules27165114 36014351 PMC9416537

[B27] Pham M. N. Nguyen T. N. Tran L. S. Nguyen Q. B. Nguyen T. H. Pham T. M. Q. (2023). epiTCR: a highly sensitive predictor for TCR-peptide binding. Bioinforma. Oxf. Engl. 39 (5), btad284. 10.1093/bioinformatics/btad284 PMC10159657 37094220

[B28] Rose P. W. Beran B. Bi C. Bluhm W. F. Dimitropoulos D. Goodsell D. S. (2011). The RCSB Protein Data Bank: redesigned web site and web services. Nucleic acids Res. 39 (Database issue), D392–D401. 10.1093/nar/gkq1021 21036868 PMC3013649

[B29] Roy A. Kucukural A. Zhang Y. (2010). I-TASSER: a unified platform for automated protein structure and function prediction. Nat. Protoc. 5 (4), 725–738. 10.1038/nprot.2010.5 20360767 PMC2849174

[B30] Rudolph M. G. Stanfield R. L. Wilson I. A. (2006). How TCRs bind MHCs, peptides, and coreceptors. Annu. Rev. Immunol. 24, 419–466. 10.1146/annurev.immunol.23.021704.115658 16551255

[B31] Schulz G. E. Schirmer R. H. (1979). Principles of protein structure. New York: Springer.

[B32] Shokrani H. Shokrani A. Seidi F. Kucińska-Lipka J. Makurat-Kasprolewicz B. Saeb M. R. (2023). Artificial intelligence for biomedical engineering of polysaccharides: a short overview. Curr. Opin. Biomed. Eng. 27, 100463. 10.1016/j.cobme.2023.100463

[B33] Shugay M. Bagaev D. V. Zvyagin I. V. Vroomans R. M. Crawford J. C. Dolton G. (2018). VDJdb: a curated database of T-cell receptor sequences with known antigen specificity. Nucleic Acids Res. 46 (D1), D419–D427. 10.1093/nar/gkx760 28977646 PMC5753233

[B34] Siva Shanmugam N. R. Jino Blessy J. Veluraja K. Gromiha M. M. (2021). Prediction of protein-carbohydrate complex binding affinity using structural features. Briefings Bioinforma. 22 (4), bbaa319. 10.1093/bib/bbaa319 33313775

[B35] Song Y. DiMaio F. Wang R. Y. Kim D. Miles C. Brunette T. (2013). High-resolution comparative modeling with RosettaCM. Structure (London, England: 1993) 21 (10), 1735–1742. 10.1016/j.str.2013.08.005 24035711 PMC3811137

[B36] Tickotsky N. Sagiv T. Prilusky J. Shifrut E. Friedman N. (2017). McPAS-TCR: a manually curated catalogue of pathology-associated T cell receptor sequences. Bioinformatics 33 (18), 2924–2929. 10.1093/bioinformatics/btx286 28481982

[B37] Tunyasuvunakool K. Adler J. Wu Z. Green T. Zielinski M. Žídek A. (2021). Highly accurate protein structure prediction for the human proteome. Nature 596, 590–596. 10.1038/s41586-021-03828-1 34293799 PMC8387240

[B38] Wang W. Peng Z. Yang J. (2022). Single-sequence protein structure prediction using supervised transformer protein language models. Nat. Comput. Sci. 2, 804–814. 10.1038/s43588-022-00373-3 38177395

[B39] Weber A. Born J. Rodriguez Martínez M. (2021). TITAN: T-cell receptor specificity prediction with bimodal attention networks. Bioinforma. Oxf. Engl. 37 (Suppl. 1), i237–i244. 10.1093/bioinformatics/btab294 PMC8275323 34252922

[B40] Winge-Main A. K. Wälchli S. Inderberg E. M. (2020). T cell receptor therapy against melanoma-Immunotherapy for the future?. Scand. J. Immunol. 92 (4), e12927. 10.1111/sji.12927 32640053

[B41] Wu R. Ding F. Wang R. Shen R. Zhang X. Luo S. (2022). High-resolution *de novo* structure prediction from primary sequence. bioRxiv [Preprint]. 10.1101/2022.07.21.500999

[B42] Xu Y. Qian X. Tong Y. Li F. Wang K. Zhang X. (2022a). AttnTAP: a dual-input framework incorporating the attention mechanism for accurately predicting TCR-peptide binding. Front. Genet. 13, 942491. 10.3389/fgene.2022.942491 36072653 PMC9441555

[B43] Xu Y. Qian X. Zhang X. Lai X. Liu Y. Wang J. (2022b). DeepLION: deep multi-instance learning improves the prediction of cancer-associated T cell receptors for accurate cancer detection. Front. Genet. 13, 860510. 10.3389/fgene.2022.860510 35601486 PMC9121378

[B44] Zhang P. Bang S. Lee H. (2023). PiTE: TCR-epitope binding affinity prediction pipeline using transformer-based sequence encoder. Pac. Symposium Biocomput. Pac. Symposium Biocomput. 28, 347–358. 10.1142/9789811270611_0032 36540990

[B45] Zhang Y. Ye T. Xi H. Juhas M. Li J. (2021). Deep learning driven drug discovery: tackling severe acute respiratory syndrome coronavirus 2. Front. Microbiol. 12, 739684. 10.3389/fmicb.2021.739684 34777286 PMC8581544

[B46] Zvyagin I. V. Tsvetkov V. O. Chudakov D. M. Shugay M. (2020). An overview of immunoinformatics approaches and databases linking T cell receptor repertoires to their antigen specificity. Immunogenetics 72 (1–2), 77–84. 10.1007/s00251-019-01139-4 31741011

